# Synthesis of [{AgO_2_CCH_2_OMe(PPh_3_)}*_n_*] and theoretical study of its use in focused electron beam induced deposition

**DOI:** 10.3762/bjnano.8.262

**Published:** 2017-12-06

**Authors:** Jelena Tamuliene, Julian Noll, Peter Frenzel, Tobias Rüffer, Alexander Jakob, Bernhard Walfort, Heinrich Lang

**Affiliations:** 1 Vilnius University, Institute of Theoretical Physics and Astronomy, Sauletekio av. 3, Vilnius, Lithuania; 2 Faculty of Natural Sciences, Institute of Chemistry, Inorganic Chemistry, D-09107 Chemnitz, Germany

**Keywords:** DFT, DSC, FEBID, silver(I) carboxylate, solid-state structure, TGA

## Abstract

The synthesis, chemical and physical properties of [{AgO_2_CCH_2_OMe}*_n_*] (**1**) and [{AgO_2_CCH_2_OMe(PPh_3_)}*_n_*] (**2**) are reported. Consecutive reaction of AgNO_3_ with HO_2_CCH_2_OMe gave **1**, which upon treatment with PPh_3_ produced **2**. Coordination compound **2** forms a 1D coordination polymer in the solid state as evidenced by single crystal X-ray structure analysis. The coordination geometry at Ag^+^ is of the [3 + 1] type, whereby the carboxylate anions act as bridging ligands. The formation of PPh_3_–Ag(I) coordinative bonds results in distorted T-shaped AgPO_2_ units, which are stabilized further by an additional O–Ag dative bond. TG and TG–MS measurements show that **1** and **2** decompose at 190–250 °C (**1**) and 260–300 °C (**2**) via decarboxylation, involving Ag–P (**2**), C–C and C–O bond cleavages to give elemental silver as confirmed by PXRD studies. In order to verify if polymeric **2** is suitable as a FEBID precursor for silver deposition, its vapor pressure was determined (*p*_170 °C_ = 5.318 mbar, *∆H_vap_* = 126.1 kJ mol^−1^), evincing little volatility. Also EI and ESI mass spectrometric studies were carried out. The dissociation of the silver(I) compound **2** under typical electron-driven FEBID conditions was studied by DFT (B3LYP) calculations on monomeric [AgO_2_CCH_2_OMe(PPh_3_)]. At an energy of the secondary electrons up to 0.8 eV elimination of PPh_3_ occurs, giving Ag^+^ and O_2_CCH_2_OMe^−^. Likewise, by release of PPh_3_ from [AgO_2_CCH_2_OMe(PPh_3_)] the fragment [AgO_2_CCH_2_OMe]^−^ is formed from which Ag^+^ and O_2_CCH_2_OMe^−^ is generated, further following the first fragmentation route. However, at 1.3 eV the initial step is decarboxylation giving [AgCH_2_OMe(PPh_3_)], followed by Ag–P and Ag–C bond cleavages.

## Introduction

Focused electron beam induced deposition (FEBID) is a cost efficient direct resist-free chemical vapor deposition technique producing free-standing 3D metal-containing nanoscale structures in a single step on, for example, surfaces of sub-10 nm size using a variety of materials with a high degree of spatial and time-domain control [1–3]. Up to now, FEBID relies on the chemical availability of chemical vapor deposition (CVD) precursors. However, such precursors are not optimized for the electron-driven FEBID process and hence molecular precursors particularly adapted to its underlying electron-induced fragmentation mechanisms are needed.

Commonly, a FEBID precursor should display a high vapor pressure at reasonable temperatures and must have a satisfactorily long residence time on the surface typically lasting micro- to milliseconds, otherwise the corresponding layer thickness will be too small [4]. The respective volatiles, as result from dissociation, should be quickly removed to avoid their entrapment in the respective deposit. Therefore, precursors must be designed, which completely decompose under typical FEBID conditions. Recently, Botman et al. highlighted the difficulty in the deposition of pure metals [5]. In this context, the deposition of, for example, silver is challenging, since there is a lack of volatile silver precursors for FEBID processes [5]. Silver (nano)structures are of importance, for example, in circuits, batteries, LED or RFID chips, medicine and photovoltaics [1].

Recently, it was shown that coordination compounds, for example, silver(I) carboxylates can successfully be applied as single-source species for silver nanoparticle formation [6] and as gas-phase precursors in the deposition of pure, dense and conformal thin silver films [7–9].

This study aims for showing if [{AgO_2_CCH_2_OMe(PPh_3_)}*_n_*] is a suitable FEBID precursor for silver deposition. Thus, we discuss the synthesis and the chemical and physical properties of [{AgO_2_CCH_2_OMe}*_n_*] and [{AgO_2_CCH_2_OMe(PPh_3_)}*_n_*]. DFT (B3LYP) studies were applied to predict the most favorable fragmentation pathways according to the lowest energy of appearance for [AgO_2_CCH_2_OMe(PPh_3_)].

## Methods of Investigation

### Experimental

The synthetic methodologies were performed under an atmosphere of argon with the solvents degassed prior to use. Ethanol (99%) was distilled from sodium/diethyl phthalate, and acetonitrile (99%) by distillation from sodium hydride and then from P_2_O_5_. Dichloromethane (95%) and diethyl ether (99%) were dried with a solvent purification system (MB SPS-800, MBraun). Silver(I) nitrate (99%), methoxyacetic acid (97%), triethylamine (99%), triphenylphosphine (99%) were obtained from commercial suppliers and used without further purification.

NMR spectra were recorded with a Bruker Advance III 500 spectrometer operating at 500.3 MHz for ^1^H, 125.7 MHz for ^13^C{^1^H} and 101.3 MHz for ^31^P{^1^H} in the Fourier transform mode at 298 K. Chemical shifts are reported in δ (ppm) downfield from tetramethylsilane with the solvent as reference signal (^1^H NMR, CDCl_3_ δ = 7.26; ^13^C{^1^H} NMR, CDCl_3_ δ = 77.16 ppm) or by external standards (^31^P{^1^H} NMR relative to 85% H_3_PO_4_ 0.0 ppm and P(OMe)_3_ 139.0 ppm).

The FTIR spectra were recorded using a Thermo Nicolet IR 200 instrument. Vapor pressure experiments were performed with a Mettler Toledo TGA/DSC1 1100 system with a UMX1 balance. The TG, DSC and TG–MS experiments were performed with a Mettler Toledo TGA/DSC1 1600 system with a MX1 balance coupled with a Pfeiffer Vacuum MS Thermostar GSD 301 T2 mass spectrometer. EI and high-resolution ESI mass spectra were recorded with a Finnigan MAT 8230 SQ instrument by using of ionization potential of 70 eV (EI) and a Bruker Daltonik micrOTOF–QII mass spectrometer (Supporting Information File 1, Figures S1–S5).

Single crystal X-ray diffraction data of **2** were collected with an Bruker Smart CCD 1k diffractometer with Mo Kα radiation (λ = 0.71073 Å) at 298 K. All structures were solved by direct methods using SHELXS-2013 and refined by full-matrix least-squares procedures on *F*^2^ using SHELXL-2013 [10]. All non-hydrogen atoms were refined anisotropically and all *C*-bonded hydrogen atoms were refined using a riding model. Graphics were created by using SHELXTL. Data have been deposited with the Cambridge Structural Database under CCDC 1552018.

**Synthesis of [{AgO_2_CCH_2_OMe}*_n_*] (1)** [11]. Silver(I) nitrate (0.5 g (2.94 mmol)) was dissolved in a mixture of 20 mL of ethanol and 0.25 mL of acetonitrile at 25 °C. To this solution a mixture of 0.26 g (2.89 mmol, 0.22 mL) of HO_2_CCH_2_OMe and 0.29 g (2.94 mmol, 0.4 mL) of NEt_3_ was dropwise added in between 5 min. During the course of the reaction **1** precipitated as a colorless solid. After 2 h of stirring at ambient temperature the supernatant solution was decanted and the thus obtained solid was thoroughly washed with cold ethanol (2 × 20 mL at 0 °C) and diethyl ether (2 × 15 mL). Yield: 0.53 g (2.69 mmol, 91%, based on HO_2_CCH_2_OMe; silver content 55%).

Anal. calcd for C_3_H_5_AgO_3_: C, 18.30; H, 2.56%; found: C, 18.45, H, 2.63%; mp 190 °C decomposition; IR (KBr, cm^−1^) υ: 2999 (m), 2835 (m), 1610 (CO_2,asym_, vs), 1585 (s), 1437 (CO_2,sym_, s), 1430 (s), 1332 (vs), 1206 (s), 1115 (vs), 990 (m), 932 (w), 769 (m).

**Synthesis of [{AgO_2_CCH_2_OMe(PPh_3_)}*_n_*] (2)** [11]. Coordination compound **1** (0.53 g, 2.69 mmol) was dissolved in 30 mL of dichloromethane and 0.69 g (2.63 mmol) of PPh_3_ were added in a single portion at 0 °C. The reaction solution was allowed to stir at ambient temperature for 2 h. Afterwards it was filtered through a pad of Celite and all volatiles were removed from the filtrate in vacuum, whereby a colorless solid remained. Yield: 1.05 g (2.29 mmol, 85% based on **1**).

Anal. calcd for C_21_H_20_AgO_3_P: C, 54.92; H, 4.39%; found: C, 54.69; H, 4.47%; mp 145 °C; IR (KBr, cm^−1^) *υ*: 3056 (w), 2977 (w), 1594 (m), 1585 (m), 1575 (CO_2,asym_, s), 1569 (s), 1479 (m), 1436 (m), 1402 (CO_2,sym_, m), 1319 (m), 1189 (m), 1104 (s), 1026 (w), 997 (w), 934 (m), 907 (w), 754 (m), 745 (m), 706 (m), 694 (s); ^1^H NMR (CDCl_3_) δ 3.44 (s, 3H, CH_3_), 4.05 (s, 2H, CH_2_), 7.40–7.50 (m, 15H, C_6_H_5_); ^13^C{^1^H} NMR (CDCl_3_) δ 59.0 (CH_3_), 71.9 (CH_2_), 129.4 (d, ^3^*J*_P,C_ = 11 Hz, ^m^C/C_6_H_5_), 130.0 (d, ^1^*J*_P,C_ = 40 Hz, ^i^C/C_6_H_5_), 131.5 (d, ^4^*J*_P,C_ = 2.0 Hz, ^p^C/C_6_H_5_), 134.1 (d, ^2^*J*_PC_ = 16 Hz, ^o^C/C_6_H_5_); ^31^P{^1^H} NMR (CDCl_3_) δ 16.0 (s, AgP(C_6_H_5_)_3_); HRMS (ESI–TOF) *m*/*z*: calcd for C_39_H_35_Ag_2_O_3_P_2_ [(Ag_2_(O_2_CCH_2_OMe)(PPh_3_)_2_]^+^, 827.0158; found, 827.0218; C_36_H_30_AgP_2_ [(Ag(PPh_3_)_2_]^+^, 631.0874; found, 631.0864; C_18_H_15_AgP [AgPPh_3_]^+^, 368.9962; found, 368.9946; EIMS *m*/*z* (%): 262 (100) [PPh_3_]^+^, 108 (42) [PPh]^+^, 369 (0.4) [AgPPh_3_]^+^.

**Crystal and structural refinement data for 2**: C_21_H_20_AgO_3_P, *M* = 459.12 g mol^−1^, space group *Cc*, λ = 9.71073 Å, *a* = 16.656(7) Å, *b* = 14.874(5) Å, *c* = 7.922(3) Å, *V* = 1960.0(13) Å^3^, *Z* = 4, δ_calcd._ = 1.556 g cm^−3^, µ = 1.127 mm^−1^, *T* = 298 K, θ range 2.449–24.989°, 6332 reflections collected, 3353 independent reflections, (*R*_int_ = 0.034), *R*_1_ = 0.0369 (all data), *wR*_2_ = 0.0624 [*I* ≥ 2σ(*I*)]. CCDC-No. 1552018.

### Theoretical studies

The molecular monomeric structure of **2** and its fragmentation behavior under typical FEBID conditions was studied by Becke’s three-parameter hybrid functional, applying the non-local correlation provided by Lee, Yang, and Parr (B3LYP) [12]. This study was performed with 3-21G for Ag and 6-31++G** for the other atoms to satisfy both accuracy of the investigations and appropriate computing time and resources. The structure parameters of [AgO_2_CCH_2_OMe(PPh_3_)] and fragments thereof have been optimized with no symmetry constraint. The vibration frequencies were examined to check the optimization results accuracy. The zero-point energy was not included in the evaluation of the appearance energy because of their difference insignificancy. The potential of fragment appearance was calculated as the difference between the total energy of the molecule and the sum of the total energies of the fragments predicted. The calculations for the final states of the compounds are presented for the case of dissociation without taking into account the activation energy of the reverse reaction (*Е*_r_). The total number of the decomposition reactions investigated was ≈50. The reactions of a dissociative ionization, dipolar dissociation, dissociative electron attachment etc. were studied to obtain the reaction pathways energetically most likely and relate to experimentally obtained species. It implies that the energetic of reactions leading from the intact neutral complex to different combinations of fragments were obtained and these results were used to deduce the most likely pathway. In this study we present only five decomposition ways, where the sum of the potential of appearance of the fragments, related to experimentally obtained species, is the lowest.

The electronegativity, chemical hardness and chemical softness were calculated as follows:


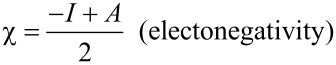



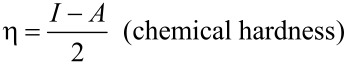



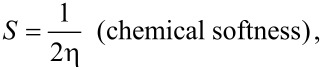


where *I* and *A* are the ionization potential and the electron affinity, respectively. The ionization potential and affinity were determined as difference of energies of ionized and neutral [AgO_2_CCH_2_OMe(PPh_3_)]. Processes, where molecular ions are formed with energies in excess of the ionization potential, may possess not sufficient energy to be decomposed according to the lowest energy pathway, were also taken into account. The calculations were performed for molecular species and not for 1D coordination polymer **2**. Gaussian program packages were applied.

## Results and Discussion

### Chemical properties

**Scheme 1:**
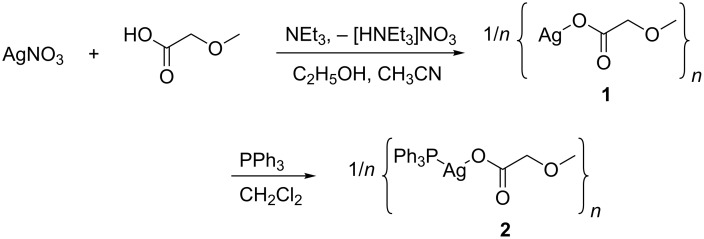
Synthesis of [{AgO_2_CCH_2_OMe}*_n_*] (**1**) and [{AgO_2_CCH_2_OMe(PPh_3_)}*_n_*] (**2**).

Coordination polymers **1** and **2** were prepared by a consecutive synthetic methodology as shown in Scheme 1. In this respect, AgNO_3_ was reacted with HO_2_CCH_2_OMe in a molar ratio of 1:1 in the presence of NEt_3_ to afford the respective silver(I) carboxylate [{AgO_2_CCH_2_OMe}*_n_*] (**1**), which gives [{AgO_2_CCH_2_OMe(PPh_3_)}*_n_*] (**2**), when treated with equimolar amounts of PPh_3_ (Scheme 1).

Coordination polymers **1** and **2** are colorless solids, which are stable towards air and moisture and hence can be safely handled under aerobe conditions. Silver carboxylate **2** shows, in comparison to **1**, an enhanced solubility in polar organic solvents such as dichloromethane and tetrahydrofuran, which is traced to the PPh_3_ ligand in **2**. In addition, this coordination compound possesses excellent light stability, since it can be stored under sun light for months. Further beneficial is that **1** and **2** do not undergo decomposition prior to evaporation with the loss or dissociation of any ligand below 150 °C.

**Figure 1:**
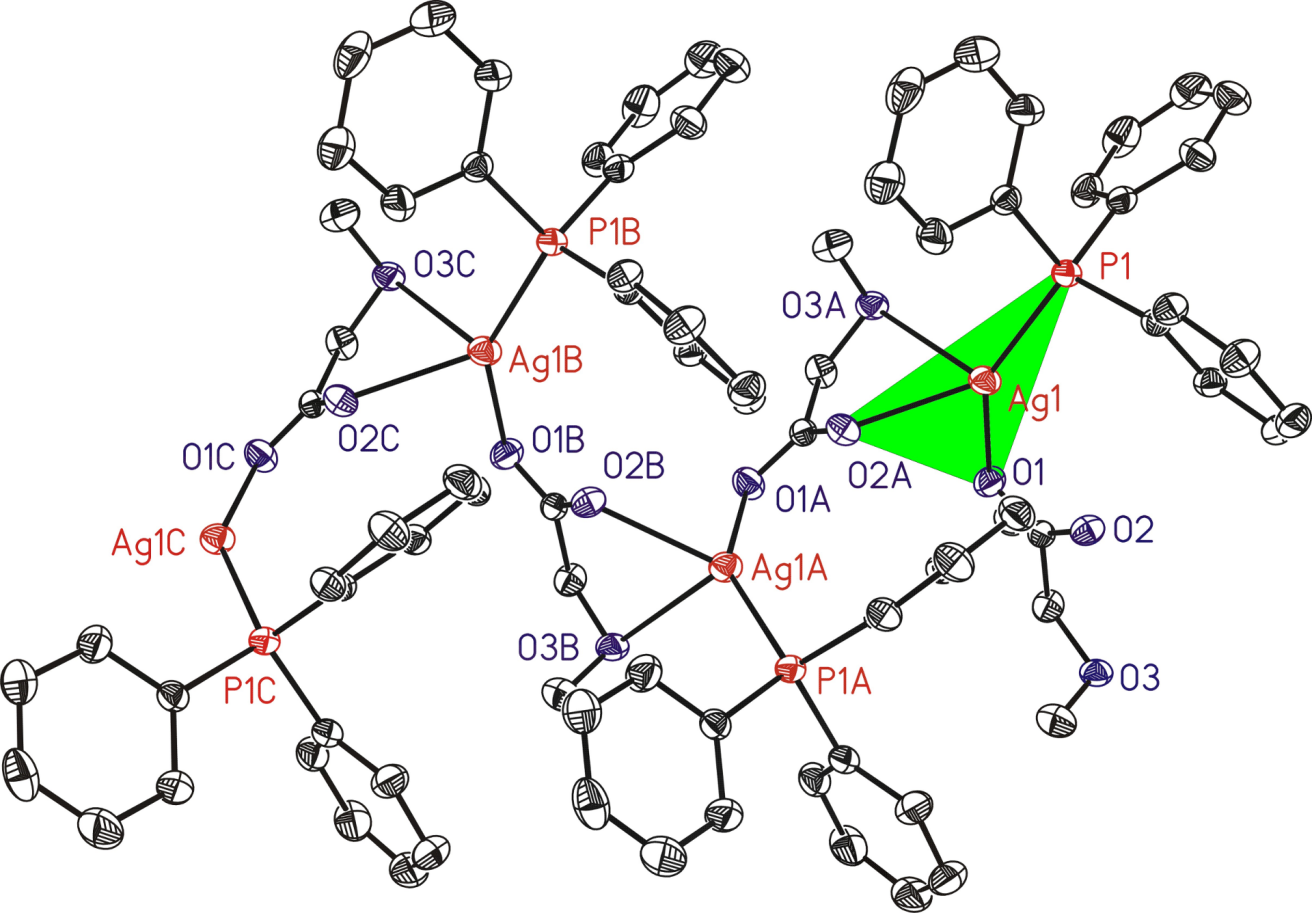
ORTEP (30% probability ellipsoids) of a selected part of the 1D coordination polymer formed by **2** in the solid state. All hydrogen atoms are omitted for clarity. The green triangle indicates the almost planar AgPO_2_ coordination units of the silver(I) ions of **2** on a selected case. Symmetry used: “A” = *x*, –*y*, –^1^/_2_ + *z*. “B” = *x*, *y*, –*z* + ^1^/_2_. “C” = *x,* –*y*, –^3^/_2_ + *z*. Selected bond lenghts (Å) and angles (°): Ag1–O1 2.269(4), Ag1–P1 2.3474(18), Ag1–O2A 2.382(4), Ag1–O3A 2.667(3) bond angles: O1–Ag1–P1 144.76(12), O1–Ag1–O2A 84.37(15), P1–Ag1–O2A 130.59(11), O1–Ag1–O3A 89.03(11), P1–Ag1–O3A 107.91(8), O2–Ag1–O3A 65.91(10).

The identity of **1** was confirmed by elemental analysis and IR spectroscopy (Experimental), while **2** was additionally characterized by ^1^H, ^13^C{^1^H} and ^31^P{^1^H} NMR (Experimental). An indication that in the silver(I) species **1** and **2** µ-bridging carboxylates are present can be deduced from the difference of the asymmetric and symmetric vibrations (∆*υ*_CO2_ = 173 cm^−1^) (Experimental). This value is close to the one obtained for the appropriate sodium salt (∆*υ*_CO2_ = 192 cm^−1^; *υ*_asym_ = 1615 cm^−1^, *υ*_sym_ = 1423 cm^−1^), confirming that in the respective carboxylate complexes the organic ligand is µ-bridging the silver atoms [13]. This bonding motif was confirmed by single crystal X-ray diffraction analysis of **2** (Figure 1).

In addition, EI and high-resolution ESI–TOF mass spectrometric investigations were carried out on **2**. In the ESI experiments (Experimental), fragments such as [Ag(PPh_3_)]^+^, [Ag(PPh_3_)_2_]^+^ and [Ag_2_(PPh_3_)_2_(O_2_CCH_2_OMe]^+^ could be detected, while EI experiments confirmed the formation of fragments such as [AgPPh_3_]^+^ of low intensity. As base peak PPh_3_^+^ was detected (Experimental; Supporting Information File 1, Figures S6 and S7). These results suggest the processing of **2** in nanoelectrospray liquid precursor injection as well as standard gas-phase FEBID [14].

Suitable single crystals of [{AgO_2_CCH_2_OMe(PPh_3_)}*_n_*] were obtained by layering a concentrated solution of dichloromethane containing **2** with diethyl ether at ambient temperature. A representative cut-off of the 1D coordination polymer formed by **2** in the solid state is shown in Figure 1, selected bond distances and angles are given in its Figure caption.

In the solid state **2** forms 1D coordination polymers (Figure 1). Thereby, the carboxylate anions act as µ-bridging ligands to link two adjacent silver(I) ions. Due to the coordination of a PPh_3_ group to Ag(I), virtually planar AgPO_2_ coordination units are observed. Planarity is revealed by the calculation of a mean plane. The average deviation from planarity amounts to 0.028 Å, while the highest deviation is observed for Ag1 with 0.048(1) Å. Furthermore, the sum of angles around Ag1 amounts to 359.8(2)°. The P–Ag–O angles (144.76(12)° and 130.59(11)°) and the O–Ag–O angle (84.37(16)°) show that the geometry of the AgPO_2_ coordination units is closer to T-shaped than to trigonal planar. Related coordination polymers of phosphane stabilized silver(I) carboxylates are scarcely reported, i.e., [AgOC(O)C_2_F_5_(PPh_3_)] [15] and [AgOAc(dppp)] [16] (dppp = diphenylphosphinopropane). In both of them the silver(I) ions form AgPO_2_ coordination units as described for **2**, although short Ag–Ag contacts between these AgPO_2_ were observed [15–16]. In case of **2** a related stabilization of the AgPO_2_ units is not observed, although the formation of an additional dative O–Ag bond is noticed (Figure 1) to give a [3 + 1] coordination setup around the silver(I) ions. This bonding motif is most likely responsible for the little volatility of **2** (see below). Compound [{AgO_2_CCH_2_OMe(PPh_3_)}*_n_*] (**2**) could be thus described as well as *catena*-poly{[triphenylphosphane-κP]-silver-µ-methoxyacetate-κO^3^, κO^1^:κO^2^}.

The thermal behavior of **1** and **2** in the solid state was studied by thermogravimetry (TG), thermogravimetry-coupled mass-spectrometry (TG–MS) and by differential scanning calorimetry (DSC).

**Figure 2:**
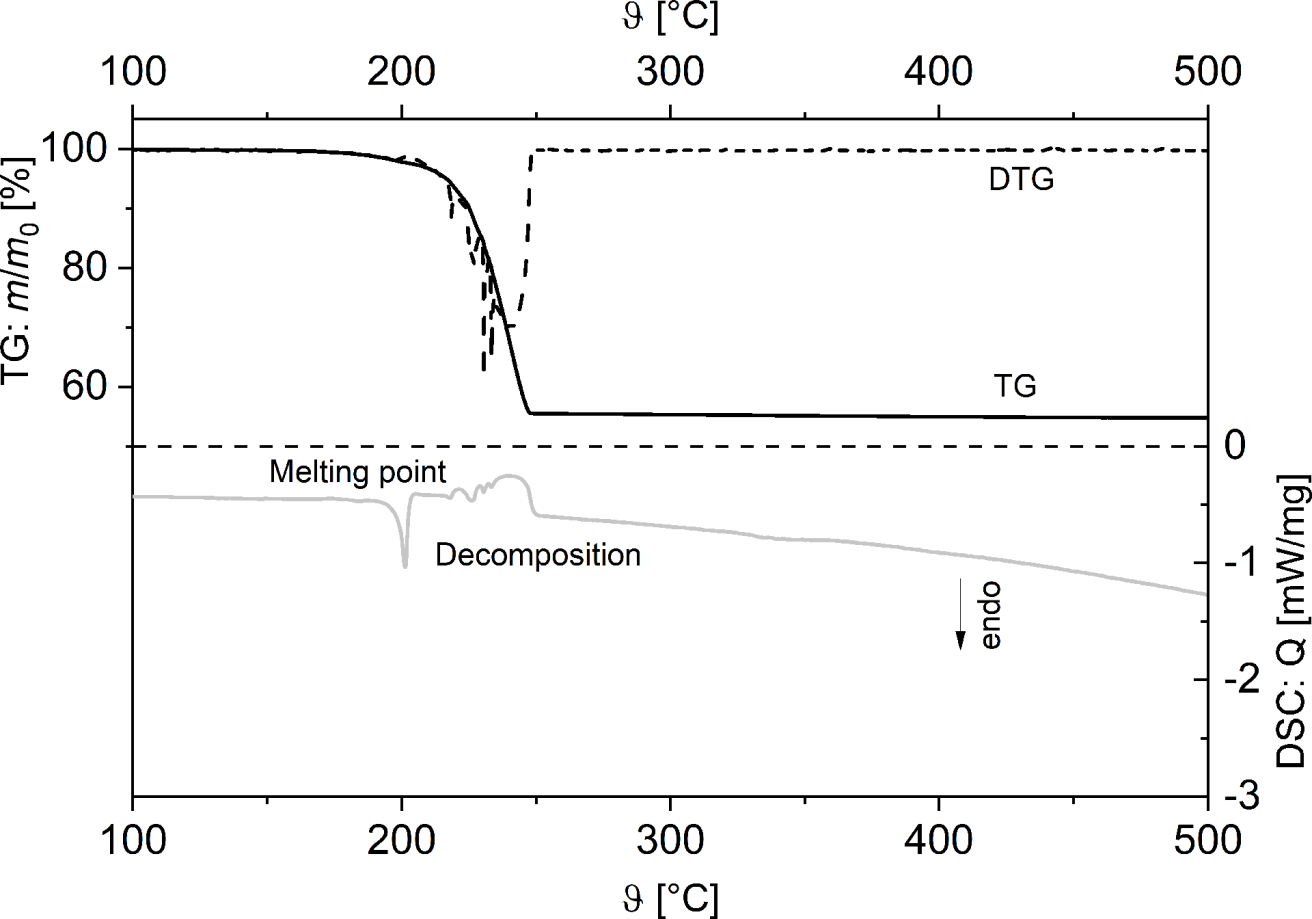
TG/DTG (top) and DSC (bottom) traces of **1** (Ar, gas flow 60 mL min^−1^, heating rate 10 °C min^−1^).

The corresponding TG/DTG and DSC traces of **1** (argon, temperature range 100–500 °C, heating rate 10 °C min^−1^) are depicted in Figure 2.

The decomposition of **1** occurs in consecutive steps, which were quantified with a mass loss of Δ*m*/*m*_0_ = 45%, matching to the formation of elemental silver (theoretical mass loss Δ*m*/*m*_0_ = 45%), which was confirmed by powder X-ray diffraction measurements (PXRD) (Supporting Information File 1, Figure S8).

The endothermic peak in the DSC study at 190 °C indicates the initial decomposition of **1**, followed by an exothermic peak at 240 °C.

**Figure 3:**
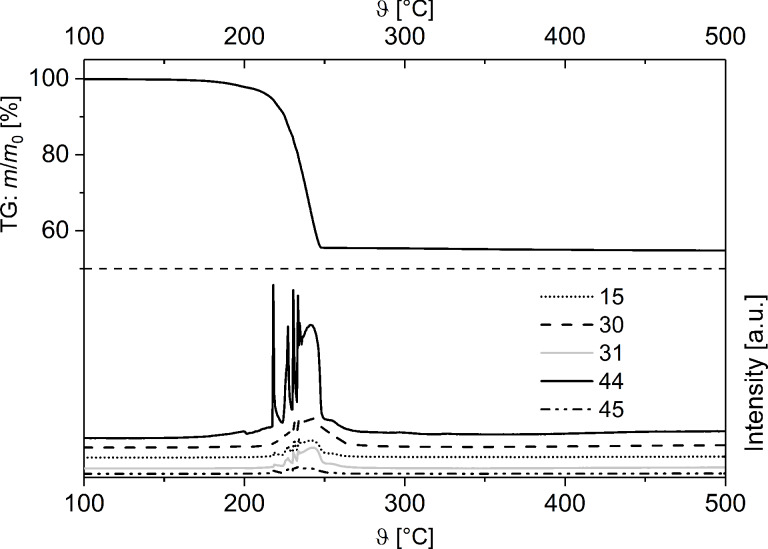
TG trace (Ar, gas flow 60 mL min^−1^, heating rate 10 °C min^−1^) and selected mass-spectrometric scans (bottom) of **1**: *m*/*z* = 45 (C_2_H_5_O^+^), 44 (CO_2_^+^), 31 (CH_3_O^+^), 30 (CH_2_O^+^), and 15 (CH_3_^+^).

In addition to TG/DTG and DSC measurements (Figure 2), TG–MS coupling experiments were carried out to gain a deeper insight into the thermal decomposition behavior of solid **1** (Figure 3).

As it can be seen from Figure 3, the decomposition of **1** occurs via elimination of carbon dioxide to give [AgCH_2_OCH_3_]. The evolution of CO_2_ is confirmed by the detection of the CO_2_^+^ fragment (*m*/*z* = 44) with high intensity. Subsequently, [AgCH_2_OCH_3_] undergoes Ag–C and C–O bond cleavages, which is verified by the observation of characteristic fragments with *m*/*z* = 45 (C_2_H_5_O^+^), 31 (CH_3_O^+^), 30 (CH_2_O^+^) and 15 (CH_3_^+^) (Figure 3). The decomposition of **1** corresponds to that one recently observed for, i.e., [AgO_2_CCH_2_(OCH_2_CH_2_)_2_OCH_3_] and [AgO_2_CCH_2_(OCH_2_CH_2_)_2_OCH_3_(PPh_3_)], respectively [6].

**Figure 4:**
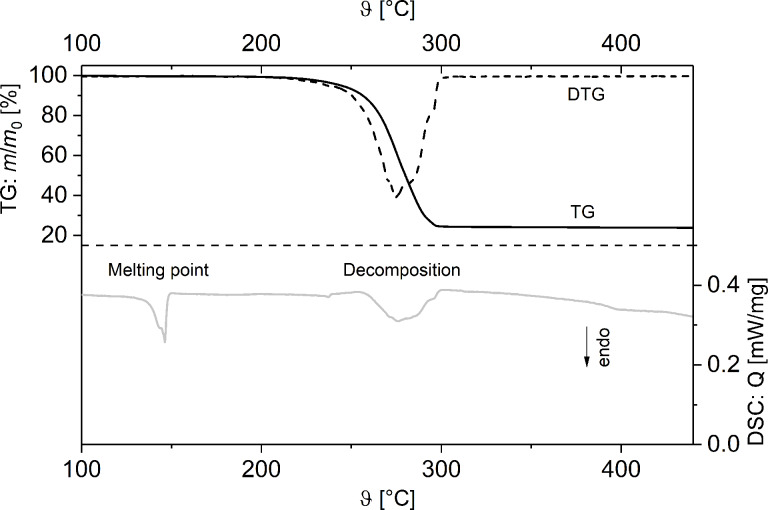
TG/DTG (top) and DSC (bottom) traces of **2** (Ar, gas flow 60 mL min^−1^, heating rate 5 °C min^−1^).

TG/DTG and DSC studies of **2** show two endothermic processes at 145 °C and 260 °C (Figure 4), of which the first one corresponds to the melting of **2**. However, weight loss of **2** during heat treatment can already be observed at 200 °C, indicating partial evaporation passing over into decomposition, whereas the corresponding DSC signals, as well as vapor pressure measurements (Supporting Information File 1, Figure S9) suggest that decomposition starts at 260 °C and is completed at 300 °C. Compared to **1**, coordination polymer **2** shows a higher decomposition temperature.

**Figure 5:**
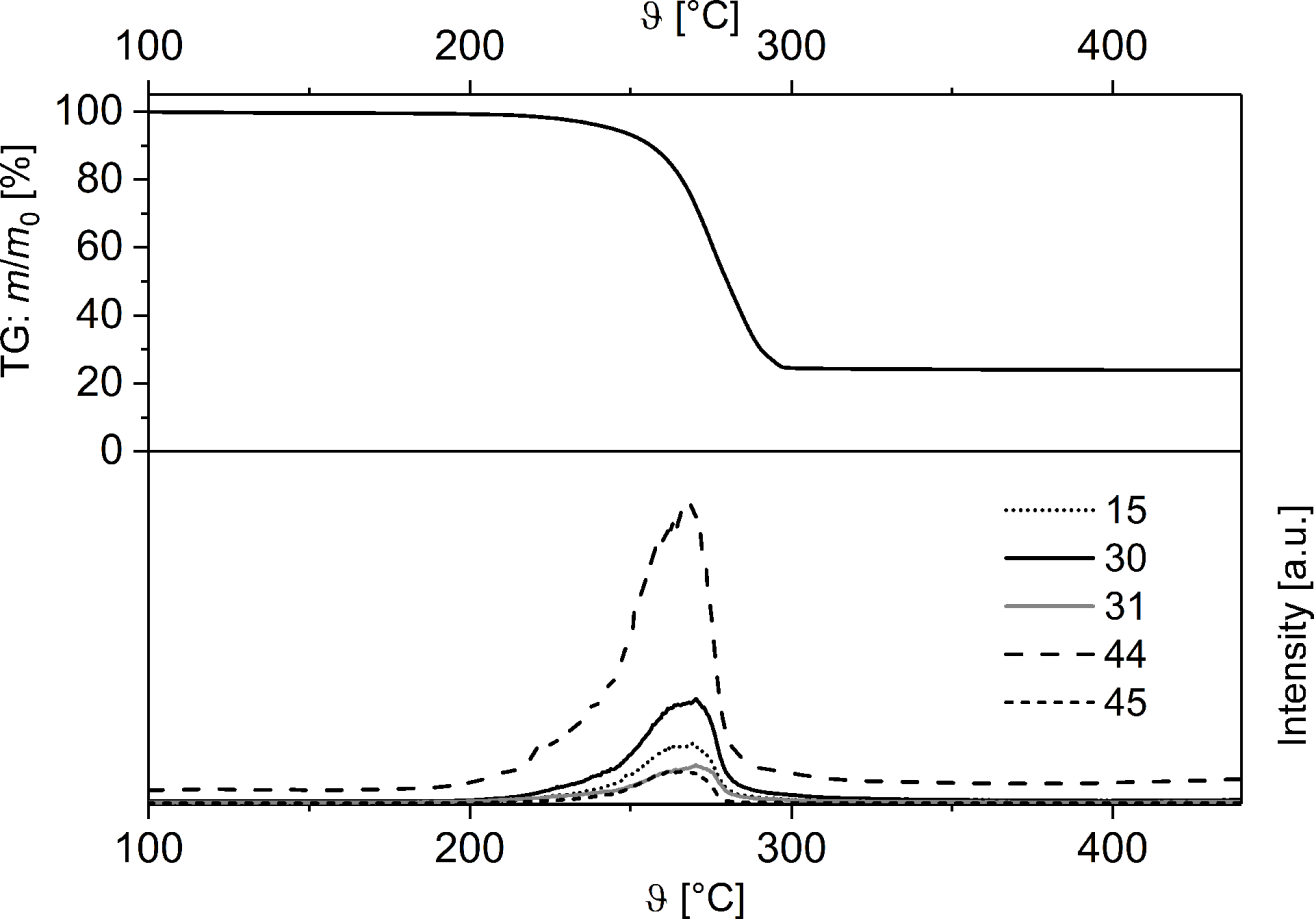
TG trace (top) (Ar, gas flow 60 mL min^−1^, heating rate 10 °C min^−1^) and selected mass-spectrometric scans (bottom) of **2**: *m*/*z* = 45 (C_2_H_5_O^+^), 44 (CO_2_^+^), 31 (CH_3_O^+^), 30 (CH_2_O^+^), and 15 (CH_3_^+^).

The remaining residue at 500 °C amounts to 23.1%, which is in agreement with the formation of elemental silver (23.5% theoretical). The formation of silver was confirmed by PXRD measurements. In addition, TG–MS coupling experiments showed fragments at *m*/*z* = 45 (C_2_H_5_O^+^), 44 (CO_2_^+^), 30 (CH_2_O^+^), 31 (CH_3_O^+^) and 15 (CH_3_^+^), indicating Ag–O, C–C and C–O bond cleavages similar to **1** (Figure 5). However, no fragments for the dative-bonded PPh_3_ group could be detected under the measurement conditions applied (Experimental, Figure 5).

**Figure 6:**
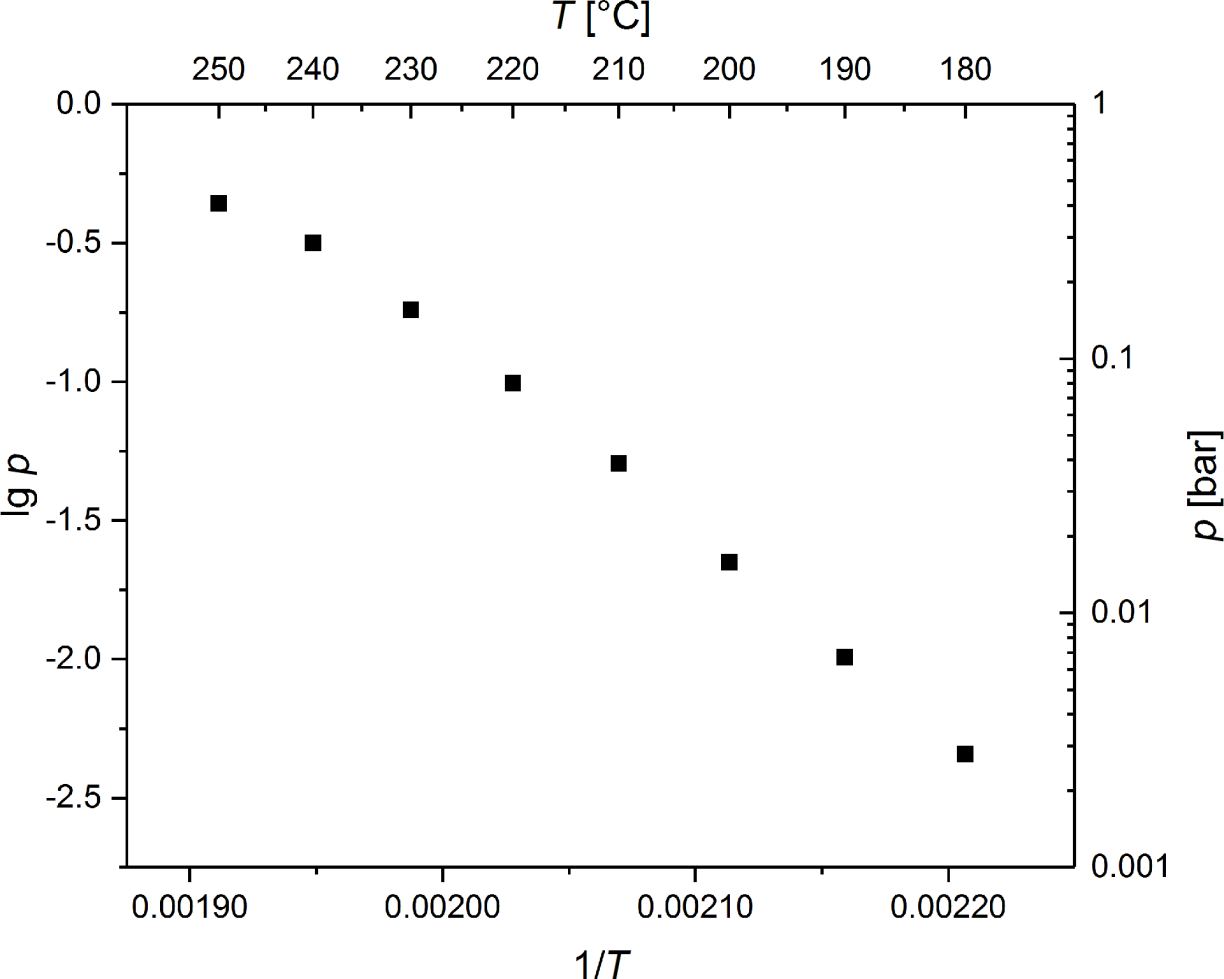
Vapor pressure of **2** (N_2_, gas flow 60 mL min^−1^).

To show, if **2** is a suitable FEBID or chemical vapor deposition (CVD) precursor for the deposition of silver, vapor pressure measurements of **2** were undertaken (Figure 6). In order to determine the volatility of **2**, a method was applied which originates from the mass-loss of the sample as a function of increasing temperature [17]. The mass-loss was defined by TG studies in an isothermal phase at different temperatures as described in [17]. The studies were carried out at atmospheric pressure under nitrogen (gas flow 60 mL min^−1^) (Figure 6). According to the TG studies, the measuring range was adjusted to 150–250 °C, so it is ensured that **2** does not decompose during the measurement (for more details see Supporting Information File 1, Figure S9). These experiments were performed thrice to provide reliable data.

The linear regression of the data is given by the characteristic Antoine parameters according to the Antoine Equation 1,

[1]
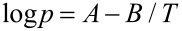


whereby A = 12.562 and B = 6574.7 with a coefficient of determination of R^2^ = 0.9951.

Based on these values the vapor pressure was determined to *P* = 5.318 mbar at 170 °C, with an evaporation enthalpy of Δ*H*_vap_ = 126.1 kJ mol^−1^.

These data approve that **2** is confined to be used as CVD precursor. In addition to the mass-spectrometric and vapor pressure measurements (see earlier), it was proven that coordination compound **2** can experimentally brought into the vapor phase. Therefore, **2** was heated to 120 °C at a pressure of 5 × 10^−2^ mbar in a Schlenk tube fitted with a sublimation finger. The corresponding sublimate was characterized by NMR and IR spectroscopy. Heating to a temperature above 260 °C gave a silver deposit.

To proof if **2** is an appropriate FEBID precursor for silver deposition under electron impact, DFT/B3LYP calculations were additionally performed on monomeric [AgO_2_CCH_2_OMe(PPh_3_)] (Experimental).

Pure metals are formed, when a chemical reaction is initiated by electrons [1], i.e., the respective precursor should be chemically stable. The calculated values of hardness and softness of monomeric **2** are equal to 3.35 eV and 0.15 eV, respectively. For comparison, silver(I) complex [Ag(hfac)(PMe_3_)] (hfac = (1,1,1,5,5,5-hexafluoropentanedionate), which was successfully used as CVD precursor for the deposition of silver, possesses values of 3.65 eV and 0.13 eV [18]. Values of the hardness >3 eV and softness <0.1 eV indicate high chemical stability and hence **2** can be considered as such, indicating reliability of the approach applied. This nicely corresponds with the experimentally observed properties of **2** (see above).

The electronic structure of monomeric **2** is similar to that of metal carbonyls [1]. For example, referring to results of our investigations, the Mulliken atomic charge of the silver atom is 0.27, which is smaller than 1.30 or 0.69 of iron or nickel atoms, consisting of the most prominent carbonyls used as precursors for FEBID [19]. In any case, the small charge on silver should be in agreement with the principle of electro-neutrality: “Stable complexes are those with structures such that each atom has only a small electric charge” [19]. On the other hand, deposition processes using complexes featuring low charged metals produce higher metal contents than highly charged metal ions [1].

One requirement for a FEBID metal precursor is that the appearance energy of the fragments formed during the dissociation process does not exceed the energy of FEBID. The relevant energy range for FEBID is 1 meV (slowed-down secondary electrons) and up to the keV regime (typical primary electron regime, forward and backscattered electrons) [19]. Hence, 50 possible fragmentation routes of mononuclear **2** under low electron impact were studied. The appearance energy *E*_ap_ was calculated for all ions shown below (2)

[2]
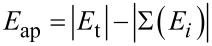


(*E*_t_ = total energy of neutral **2**, *E_i_* = total energy of all fragments produced during dissociation).

However, this calculation does not take the activation energy of the molecular ion fragmentation into account. Subsequently, only the most favorable fragmentation routes according to the lowest energy pathways are considered, which are pathways A–C.

### Fragmentation according to pathway A

The energies of the fragments formed during the decomposition of **2** concerning pathway A are summarized in Table 1 and Table 2. Here and further, the total charge of the compound consisting of isolated fragments is given. In some cases the additional analysis of the Mulliken charge of the compound was performed to exhibit which fragment carries the positive, negative or neutral charge.

**Table 1:** Appearance energy (Equation 2) of differently charged Ag and Ph_3_P/O_2_CCH_2_OMe fragments from dissociation of mononuclear **2**.

Ag charge	Ph_3_P/O_2_CCH_2_OMe charge^a^	*E*_ap_ [eV]

−1	−1	0.79
−1	0	3.82
−1	1	8.37
0	−1	1.48
0	0	4.52
0	1	9.07
1	−1	8.29
1	0	11.32
1	1	15.87

^a^Ph_3_P/O_2_CCH_2_OMe charge indicates the total charge of the compound consisting of Ph_3_P and O_2_CCH_2_OMe fragments.

**Table 2:** Appearance energy (Equation 2) of differently charged O_2_C and CH_2_OMe fragments from dissociation of O_2_CCH_2_OMe^−^.

CO_2_ charge	CH_2_OMe charge	*E*_ap_ [eV]

0	−1	1.37
0	0	1.21
0	1	8.25
1	−1	15.23
1	0	15.07
1	1	22.11

From Table 1 it can be seen that the formation of negatively charged Ph_3_P/O_2_CCH_2_OMe^−^ and negatively charged Ag^−^ requires the smallest energy of all investigated cases. The fragmentation concerning Ag(0) formation, according to the value of the appearance energy, requires 1.48 eV, which is ≈0.7 eV higher compared to the most favorable decomposition process (pathway A). Mulliken charge analysis indicates that the negative charge mostly residues on the O_2_CCH_2_OMe fragment and hence the following process is most likely


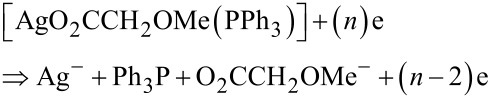


Hence, for the process 0.79 eV are necessary to produce a negatively charged Ag^−^ ion. However, the decarboxylation of negatively charged O_2_CCH_2_OMe^−^ could occur as alternative reaction route (decomposition pathways B and C). In this respect, the energy of the variously charged CO_2_ and CH_2_OMe fragments were determined, showing that at least 1.21 eV are necessary for the decarboxylation of the O_2_CCH_2_OMe^−^ fragment (Table 2). In summary, referring to the calculated data (Table 2), the most favorable fragmentation process, according to pathway A, requires at least 0.79 eV for the formation of Ag^−^ and at least 1.21 eV for the decarboxylation of the negatively charged O_2_CCH_2_OMe^−^ fragment.

### Fragmentation according to pathway B

The formation of [AgO_2_CCH_2_OMe] from neutral mononuclear **2** requires less energy than the removal of Ag from mononuclear **2** (Table 3). It was found that the development of the negatively charged [AgO_2_CCH_2_OMe]^−^ fragment is most favorable, which is based on the appearance energy, which is with 0.44–0.55 eV (Table 3) very low. Hence, the electron-induced decomposition processes can be formulated as


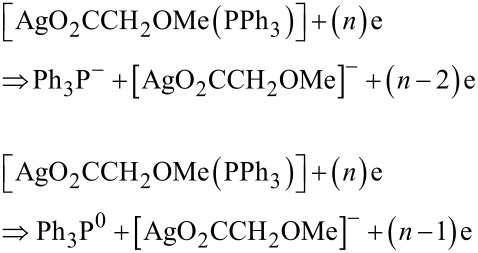


However, release of Ag from [AgO_2_CCH_2_OMe]^−^ requires at least 0.75 eV (Table 4).

**Table 3:** Appearance energy (Equation 2) of differently charged Ph_3_P and [AgO_2_CCH_2_OMe] fragments from dissociation of monomeric **2**.

Ph_3_P charge	Ag(O_2_CCH_2_OMe) charge	*E*_ap_ [eV]

−1	−1	0.55
−1	0	1.77
−1	1	9.97
0	−1	0.44
0	0	1.66
0	1	9.87
1	−1	7.42
1	0	8.64
1	1	16.84

**Table 4:** Appearance energy (Equation 2) of differently charged Ag and O_2_CCH_2_OMe fragments from dissociation of [AgO_2_CCH_2_OMe]^−^.

Ag charge	O_2_CCH_2_OMe charge	*E*_ap_ [eV]

−1	−1	0.75
−1	0	3.26
−1	1	10.11
0	−1	1.45
0	0	3.96
0	1	10.81
1	−1	8.25
1	0	10.76
1	1	17.61

Based on the data obtained (Table 4), the most favorable decomposition process is


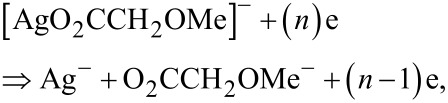


which agrees with pathway A, i.e., the building of Ag^−^ and O_2_CCH_2_OMe^−^, respectively. From negatively charged O_2_CCH_2_OMe^−^ CO_2_ is released. For this decomposition path the Ag appearance energy, according to the lowest energy pathways, is equal to 1.19 or 1.30 eV. It is larger than the *E*_ap_ of anionic Ag^−^ directly from [AgO_2_CCH_2_OMe(PPh_3_)].

In conclusion, the decomposition of mononuclear **2** under FEBID conditions occurs preferably via pathway A, when compared to B.

### Fragmentation accordingly to pathway C

The investigation of the fragmentation according to pathway C indicates that decarboxylation of mononuclear **2** requires at least 0.8 eV (Table 5).

**Table 5:** Appearance energy (Equation 2) of differently charged [AgCH_2_OMe(PPh_3_)] and CO_2_ fragments from dissociation of monomeric **2**.

CO_2_ charge	Ag(CH_2_OMe)(Ph_3_P) charge	*E*_ap_ [eV]

0	−1	1.18
0	0	0.80
0	1	6.90
1	−1	15.50
1	0	14.67
1	1	20.77

Due to the lowest *E*_ap_ (Equation 2), the formation of the neutral CO_2_ and [AgCH_2_OMe(PPh_3_)] fragments are the most favorable ones. It is interesting to note that the elimination of PPh_3_ from [AgCH_2_OMe(PPh_3_)] is a spontaneous reaction as indicated by the negative values of the free Gibbs energy (Table 6), allowing to predict following most favorable processes


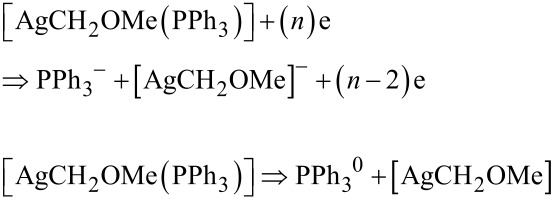


The appearance energy of negatively charged and neutral [AgCH_2_OMe], amounts to 1.45 and 1.34 eV, respectively.

**Table 6:** Appearance energy (Equation 2) and free Gibbs energy of differently charged Ph_3_P and AgCH_2_OMe fragments from dissociation of [AgCH_2_OMe(PPh_3_)].

Ph_3_P charge	AgCH_2_OMe charge	*E*_ap_ [eV]	Free Gibbs energy [kcal/mol]

−1	−1	1.45	−2.35
−1	0	1.82	8.09
−1	1	8.66	164.59
0	0	1.34	−0.89
0	1	1.72	9.55
0	0	8.55	166.05
1	1	8.32	160.57
1	0	8.69	171.01
1	−1	15.53	327.52

*E*_ap_ of Ag^−^ is smaller than those of neutral or positively charged silver (Table 7 and Table 8), forming from both the negatively charged or neutral [Ag(CH_2_OMe)] fragments.

**Table 7:** Appearance energy (Equation 2) of differently charged Ag and CH_2_OMe fragments from dissociation of [AgCH_2_OMe]^−^.

Ag charge	CH_2_OMe charge	*E*_ap_ [eV]

−1	−1	1.80
−1	0	1.64
−1	1	8.68
0	−1	2.50
0	0	2.34
0	1	9.38
1	−1	9.30
1	0	9.14
1	1	16.18

**Table 8:** Appearance energy (Equation 2) of differently charged Ag and CH_2_OMe fragments from dissociation of [AgCH_2_OMe].

Ag charge	CH_2_OMe charge	*E*_ap_ [eV]

−1	−1	1.43
−1	0	1.27
−1	1	8.31
0	−1	2.13
0	0	1.97
0	1	9.01
1	−1	8.93
1	0	8.77
1	1	15.81

The comparison of *E*_ap_ of Ag^−^ allows to state that the most favorable fragmentation of [AgCH_2_OMe], according to the lowest energy pathway, is


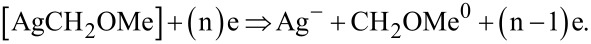


The formation of the negatively charged ion Ag^−^ is the result of the most favorable dissociation reaction, according to the lowest energy pathways A and B. The total appearance energy of Ag is equal to 3.41–3.52 eV, which is much larger than those of pathways A and B. Hence, both the A and B pathways can be realized, when the energy range for FEBID should be up to 0.8 eV, while pathway C is more favored, when the energy of the secondary electrons will be 1.3 eV.

## Conclusion

The consecutive synthesis of [{AgO_2_CCH_2_OMe(PPh_3_)}*_n_*] (**2**) by the reaction of AgNO_3_ with the carboxylic acid HO_2_CCH_2_OMe and treatment of thereby formed [{AgO_2_CCH_2_OMe}*_n_*] (**1**) with PPh_3_ is reported. The chemical, thermal and structural properties of **2** are reported. Complex **2** forms a 1D coordination polymer in the solid state in which the silver(I) ions are involved in distorted T-shaped planar AgPO_2_ coordination units, which are additionally stabilized by an O–Ag dative bond. The thermal initiated decomposition of **1** and **2** in the solid state was determined by thermogravimetry and differential scanning calorimetry. It was found that **2** possesses a somewhat higher onset temperature as **1**, which is related to the different structure of both complexes. The mechanism of dissociation for both species was studied using thermogravimetry-coupled mass spectrometry confirming that **1** decarboxylates at first followed by Ag–C, C–C and C–O bond cleavages. The remaining residue was characterized as elemental silver by PXRD. Also, coordination polymer **2** most likely decomposes by elimination of CO_2_ and PPh_3_ and the thus formed [AgCH_2_OMe] fragment dissociates as discussed for **1**. Vapor pressure measurements showed that **2** is little volatile, despite its polymeric structure, while **1** cannot be evaporated. Also EI and ESI mass spectrometric studies and sublimation experiments were carried out with **2**, indicating its usability in nanoelectrospray liquid precursor injection [14] as well as in standard gas-phase FEBID processes for silver deposition.

Based on the chemical and physical properties of **2** and on the appearance energy of silver, DFT (B3LYP) calculations were carried out to determine the decomposition pathways for mononuclear [AgO_2_CCH_2_OMe(PPh_3_)]. Three lowest energy dissociation routes exist: (i) Pathway A: Electron-induced decomposition of mononuclear **2** occurs via the formation of Ag^−^, O_2_CCH_2_OMe^−^ and Ph_3_P. From O_2_CCH_2_OMe^−^, CO_2_ is released. Pathway B: Elimination of PPh_3_ takes place at first affording [AgO_2_CCH_2_OMe]^−^ from which the fragments Ag^−^ and O_2_CCH_2_OMe^−^ occur. Pathway C: Decarboxylation of mononuclear **2** takes place producing [AgCH_2_OMe(PPh_3_)], which afterwards releases PPh_3_ forming [AgCH_2_OMe], which further decomposes to Ag. Both the A and B pathways are most favored, when the energy range for FEBID is up to 0.8 eV, while pathway C is preferred, when the energy of the secondary electrons will be up 1.3 eV.

## Supporting Information

File 1 Additional figures and tables.
