# Supplementary material for: Synthesis of [{AgO2CCH2OMe(PPh3)}n] and theoretical study of its use in focused electron beam induced deposition
Source: Beilstein J Nanotechnol. 2017 Dec 6;8:2615–24. doi: 10.3762/bjnano.8.262 (PMC5727776; doi:10.3762/bjnano.8.262)
Supplement: File 1 — Additional figures and tables. [file Beilstein_J_Nanotechnol-08-2615-s001.pdf]

Supporting Information

for

Synthesis of [{$AgO_2CCH_2OMe(PPh_3)$}$_n$] and theoretical study of its use in focused electron beam induced deposition

Jelena Tamuliene*[1,§], Julian Noll[2], Peter Frenzel[2], Tobias Rüffer[2], Alexander Jakob[2], Bernhard Walfort[2] and Heinrich Lang*[2,¶]

Address: [1]Vilnius University, Institute of Theoretical Physics and Astronomy, Sauletekio av. 3, Vilnius, Lithuania and [2]Faculty of Natural Sciences, Institute of Chemistry, Inorganic Chemistry, D-09107 Chemnitz, Germany

Email: Jelena Tamuliene - Jelena.Tamuliene@tfai.vu.lt;

Heinrich Lang - heinrich.lang@chemie.tu-chemnitz.de

*Corresponding author

[§] Phone +370 5-223-34644; Fax +370 5-2125361

[¶] Phone +49 371-531-21210; Fax +49 371-531-21219

Additional figures and tables

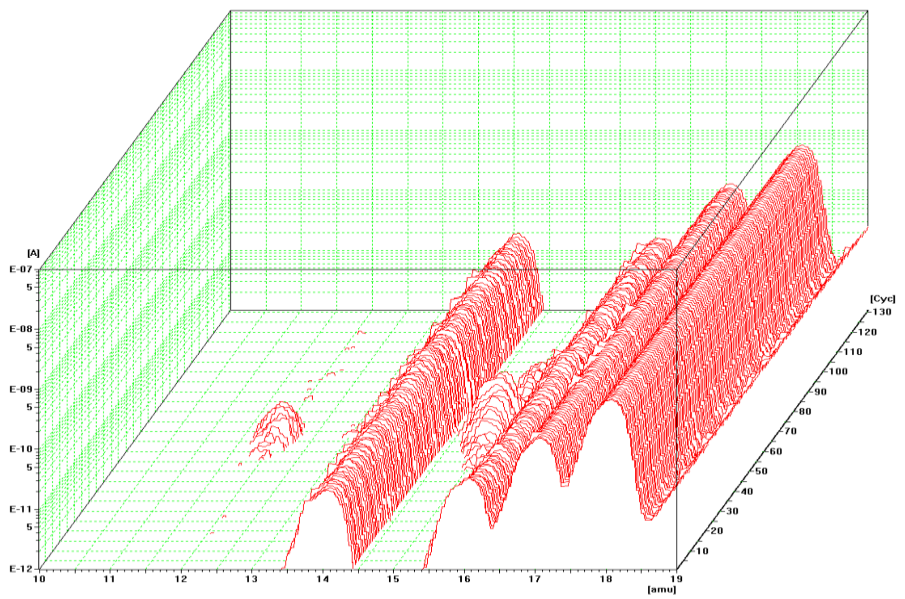

Figure S1: Selected masses (m/z = 10–19) of a time-resolved MS spectra during TG measurements of **2**.

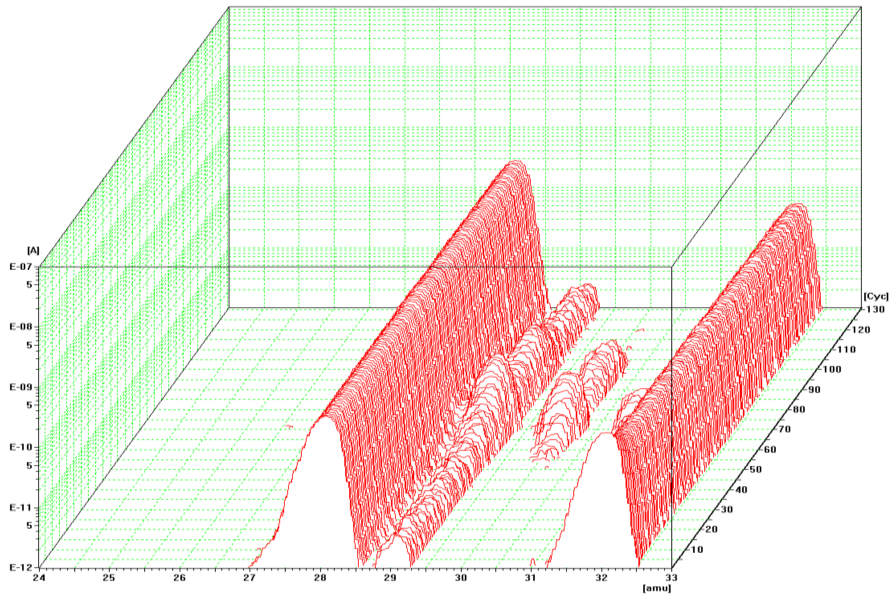

Figure S2: Selected masses (m/z = 24–33) of a time-resolved MS spectra during TG measurements of **2**.

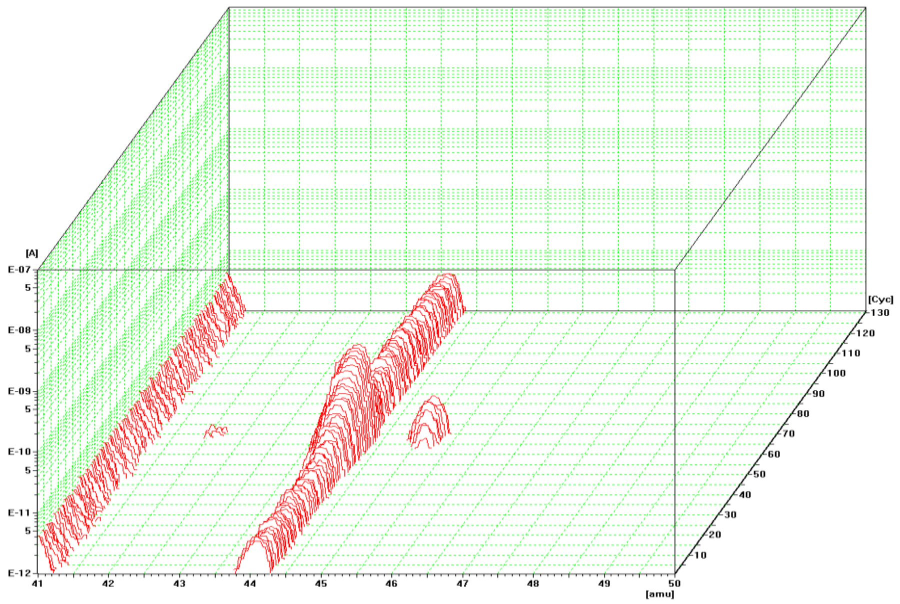

Figure S3: Selected masses (m/z = 41–50) of a time-resolved MS spectra during TG measurements of **2**.

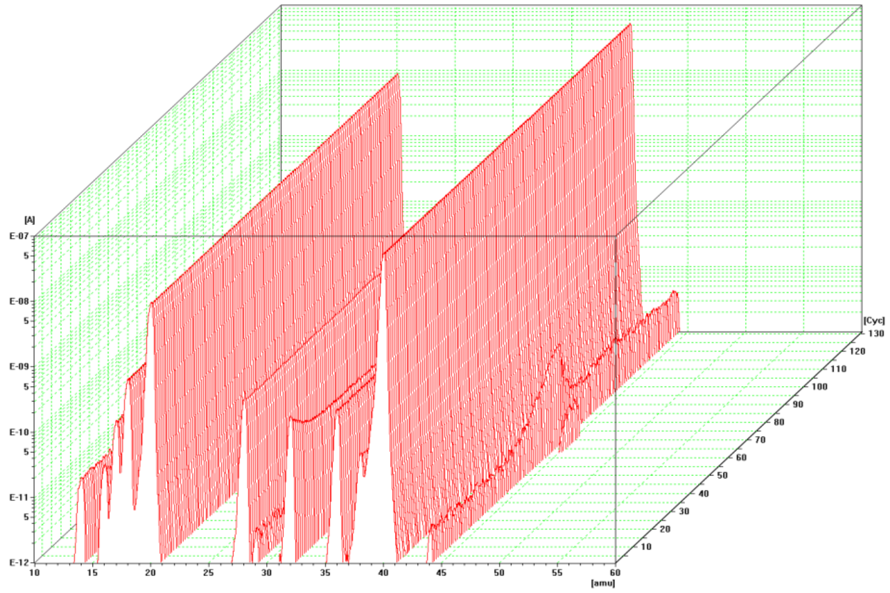

Figure S4: Selected masses (m/z = 10–60) of a time-resolved MS spectra during TG measurements of **2**.

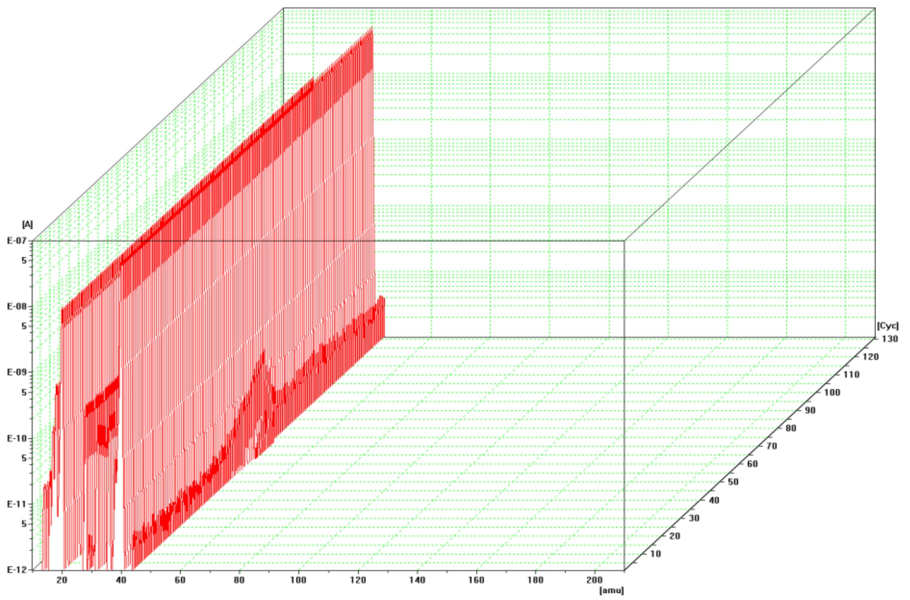

Figure S5: Selected masses (m/z = 10–210) of a time-resolved MS spectra during TG measurements of **2**.

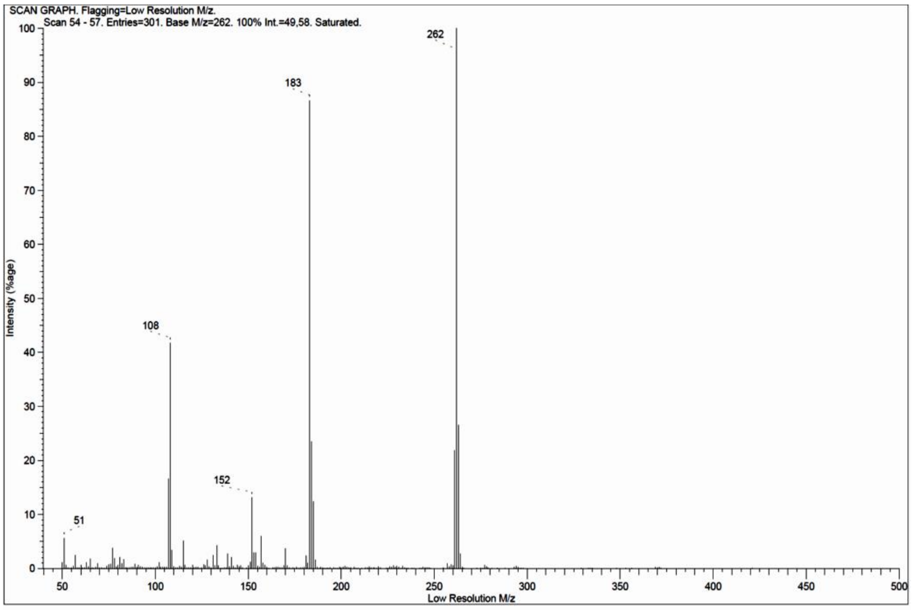

Figure S6: EI-MS spectra of **2** (m/z = 40–500).

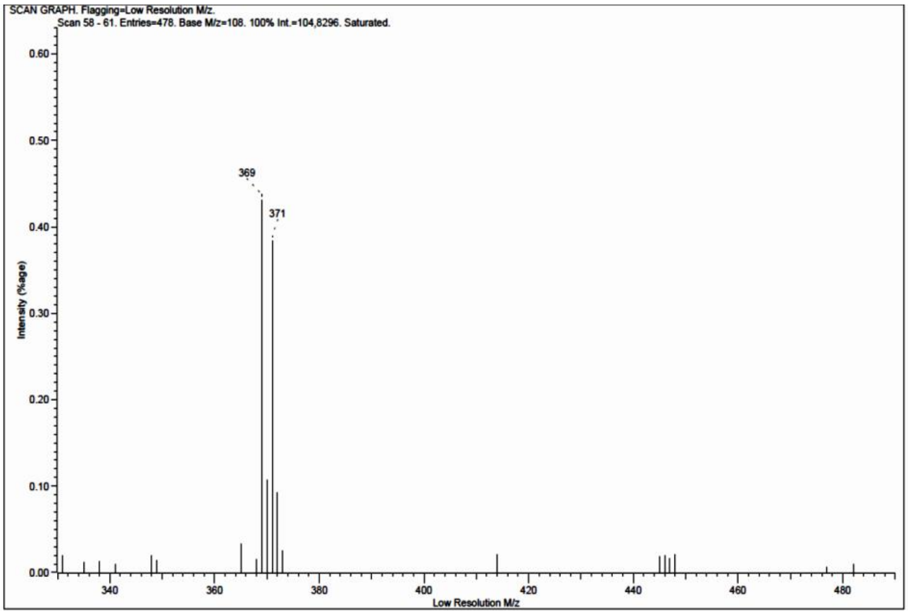

Figure S7: EI-MS spectra of **2** (*m*/*z* = 330–490).

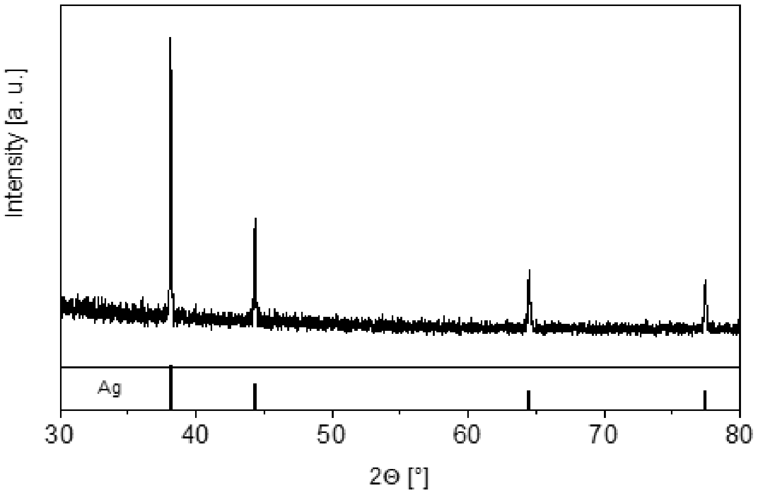

Figure S8: PXRD pattern of elemental silver after heat treatment of **1** (500 °C) with reflections of silver [ICDD: 03-065-2871].

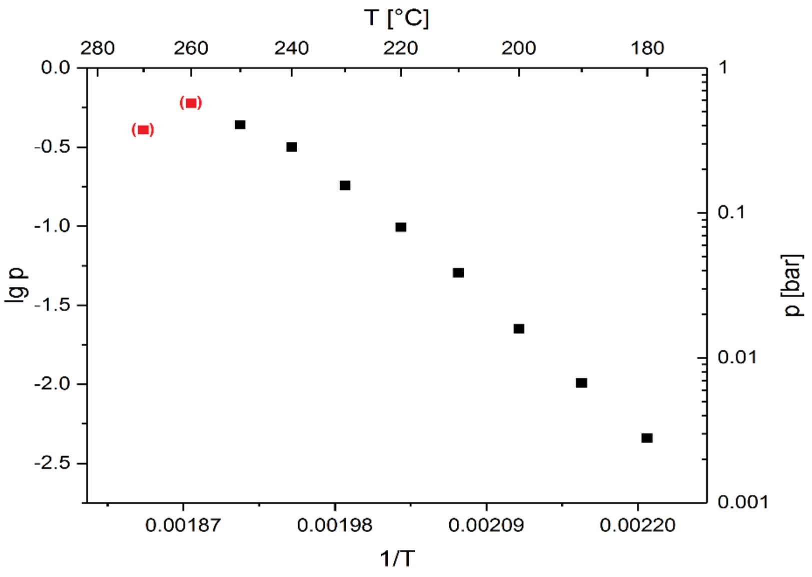

Figure S9: Vapor pressure of **2** (N_2, gas flow 60 mL min^{-1}). Red marked measurement points imply decomposition of **2**.
